# Identifying the Genetic Variation of Gene Expression Using Gene Sets: Application of Novel Gene Set eQTL Approach to PharmGKB and KEGG

**DOI:** 10.1371/journal.pone.0043301

**Published:** 2012-08-14

**Authors:** Ryan Abo, Gregory D. Jenkins, Liewei Wang, Brooke L. Fridley

**Affiliations:** 1 Division of Clinical Pharmacology, Department of Molecular Pharmacology and Experimental Therapeutics, Mayo Clinic, Rochester, Minnesota, United States of America; 2 Department of Health Sciences Research, Mayo Clinic, Rochester, Minnesota, United States of America; University of California, Irvine, United States of America

## Abstract

Genetic variation underlying the regulation of mRNA gene expression in humans may provide key insights into the molecular mechanisms of human traits and complex diseases. Current statistical methods to map genetic variation associated with mRNA gene expression have typically applied standard linkage and/or association methods; however, when genome-wide SNP and mRNA expression data are available performing all pair wise comparisons is computationally burdensome and may not provide optimal power to detect associations. Consideration of different approaches to account for the high dimensionality and multiple testing issues may provide increased efficiency and statistical power. Here we present a novel approach to model and test the association between genetic variation and mRNA gene expression levels in the context of gene sets (GSs) and pathways, referred to as gene set – expression quantitative trait loci analysis (GS-eQTL). The method uses GSs to initially group SNPs and mRNA expression, followed by the application of principal components analysis (PCA) to collapse the variation and reduce the dimensionality within the GSs. We applied GS-eQTL to assess the association between SNP and mRNA expression level data collected from a cell-based model system using PharmGKB and KEGG defined GSs. We observed a large number of significant GS-eQTL associations, in which the most significant associations arose between genetic variation and mRNA expression from the same GS. However, a number of associations involving genetic variation and mRNA expression from different GSs were also identified. Our proposed GS-eQTL method effectively addresses the multiple testing limitations in eQTL studies and provides biological context for SNP-expression associations.

## Introduction

Establishing genetic variation (e.g., single nucleotide polymorphisms (SNPs)) associated with variation in mRNA gene expression is a key component to further understand the molecular basis of human traits, including complex disease and response to drug therapies. The genetics of human mRNA expression level has been extensively studied and many mRNA expression regulatory loci or expression quantitative trait loci (eQTL) have been identified using a variety approaches, often based on the use of cell line model systems [1]. Yet, additional research in this area is needed to fully characterize and understand the mechanisms by which eQTLs regulate mRNA gene expression. A basic understanding regarding the locations of eQTLs relative to the genes in which they regulate has been established. A *cis*-acting eQTL, or *cis*-eQTL, describes a DNA sequence variant located within or outside the gene transcription unit up to a couple mega-bases away [2], [3], while *trans*-acting eQTLs, or *trans*-eQTLs, are considered to be located much further from the associated transcription unit. *Trans*-eQTLs that are associated with many mRNA gene expressions are termed “hotspots” or “master regulators”, and are presumed to influence many biological functions [4]. Mapping eQTLs in humans could help to identify the functional loci contributing to variation in human traits and has been applied to the study of many complex traits, such as asthma [5], type 2 diabetes [6], adult height [7], Crohn's disease [8], and celiac disease [9].

Identification of eQTLs in humans has been performed using analytical methods previously developed for disease-risk genetic studies by treating each mRNA gene expression level as a quantitative trait with linkage analysis methods for family-based data [10]–[13] and association analysis methods for unrelated individuals [14]–[17]. More recently, the rapid development and cost reduction of genomic arrays to capture genome-wide single nucleotide polymorphism (SNP) and mRNA expression data have resulted in the use of genome-wide association (GWA) analyses using independent samples [18]–[22]. The eQTL mapping approach with genome-wide data involves assessing the association between all possible SNP-expression pairs. These eQTL GWA studies have resulted in a large number of expression associated SNPS (eSNPs) [21], [23], [24]. The success of eQTL association mapping methods as compared to disease-risk studies may be due to the strength of eQTL signals and lack of phenotype heterogeneity; however, there are much greater multiple testing issues to consider with eQTL association mapping and therefore, a possible substantial loss in statistical power to detect the weaker associations.

A recent approach to reduce multiple testing and improve inference in genomic association analysis involves the consolidation of SNPs or expression probes into sets of related genes [i.e., gene sets (GS)], followed by a determination if the gene set is associated with a trait [25], [26]. Gene set analysis (GSA) was initially proposed for microarray expression data as a Gene Set Enrichment Analysis (GSEA) [6], [27]. The GSEA method made use of a priori biological knowledge of genes to determine the GSs, such as biochemical pathways. While many GSA methods for expression have been developed [28], recent GSA methods for expression studies have been extended for use with genome-wide SNP data [25], [29]. GSA methods designed for expression and SNP data fall into two separate categories, competitive or self-contained, based on the null hypothesis tested and within each category, methods differ widely in the statistics used for the GSs and how to assess the significance of these statistics [26], [29]. A common feature among most of the methods developed for GSA is the use of databases to define the GSs. These databases usually group genes that fall into a biological pathway or have similarly defined characteristics. A number of databases exist with different approaches and definitions for grouping genes, such as Gene Ontology [30], KEGG [31], and PharmGKB [32].

A number of recent efforts have been applied the GS enrichment methodology towards identifying eQTLs. While this strategy provides a reasonable follow up analysis to the SNP-expression pair-wise analyses, it still requires the exhaustive pair-wise tests to be performed and the necessary permutations for unbiased association testing [33], [34]. In particular, Li et al. proposed a method in which the eQTL p-values within a GS are combined using Fisher's method, followed by approximation of the distribution of the test statistic under the null hypothesis using Satterwhite's approximation [35]. An alternative approach to methods based on summary statistics (i.e., p-values) is one in which the association of SNP genotypes with mRNA gene expression levels within a given pathway is assessed with using multivariate model [36]. Examining eQTLs in the context of the Protein Interaction Network has also been done [37].

In addition to the use of GS analysis method for reducing the dimensionality of genomic data, the use of principal components analysis (PCA) has also been used in the analysis of high dimensional genomic data as a means to extract the features (e.g., components) with the most variation. The selected subset of principal components (PCs) accounting for a majority of the overall variation observed in the genomic data can then be analyzed in a manner similar to the original data. Gauderman et al. introduced the use of PCA for assessing the association of multiple SNPs within a candidate gene [38].

In this paper, we present a new approach to identify genetic variation associated with the mRNA expression by modeling SNP and mRNA expression variables within the context of pre-defined GSs. This method, referred to as gene set eQTL (GS-eQTL), is illustrated using data from a cell line model system [39]–[41] and the GSs (or pathways) defined in PharmGKB (http://www.pharmgkb.org/) [32] and KEGG (http://www.genome.jp/kegg/) [31]. Application of GS-eQTL to these two sets of GSs enable us to detect 28,597 GS-eQTL associations with an empirical false discovery rate (FDR) less than 0.05 (436 GS-eQTLs in PharmGKB and 28161 GS-eQTLs in KEGG). Replication of two of these top GS-eQTL associations using data in HapMap was also completed resulting in GS-eQTL p-values <0.05 (e.g., replication of the GS-eQTL).

In summary, our proposed approach has demonstrated its applicability and potential for analyzing the associations between SNP and mRNA expression data beyond the traditional single marker eSNP analyses. The use of GSs reduces the multiple testing and focuses on biologically relevant hypotheses. The current study, involving cell line data and PharmGKB and KEGG GSs, illustrates these two attractive features. Such methods and subsequent findings will become increasingly important in aiding the functional translation of disease risk or pharmacogenomic association findings.

## Materials and Methods

### Cell Line Model System

EBV-transformed lymphoblastoid cell lines (LCLs) from 96 African-American (AA), 96 Caucasian-American (CA), and 96 Han Chinese–American (HCA) unrelated subjects (sample sets HD100AA, HD100CAU, HD100CHI) were purchased from the Coriell Cell Repository. NIGMS collected and anonymized the samples, and all subjects provided written consent for their experimental use.

DNA from the LCLs was genotyped using Illumina HumanHap 550 K and 510 S BeadChips, which assayed 561,298 and 493,750 SNPs, respectively. Genotyping was performed in the Genotype Shared Resource at the Mayo Clinic. The genotyping data had been described previously [39]–[41]. SNP quality control procedures consisted of removal of SNPs with low call rate (<95%), low minor allele frequency (MAF) (<0.05), and departures from Hardy Weinberg Equilibrium (p<0.001). Subjects with call rates <95% were also removed from the analysis. SNP genotypes were coded in terms of the number of minor alleles (e.g., 0, 1 or 2) (i.e., additive genetic model). Missing genotypes were imputed with the mean dosage value for the SNP. Population stratification was assessed due to the use of cell lines representing multiple races/ethnic groups, as discussed in Li et al. [40] and Niu et al. [41], in which an eigen analysis was used to detect and adjust for population stratification [42].

Total RNA was extracted the cell lines using Qiagen RNeasy Mini kits (QIAGEN, Inc.). RNA quality was tested using an Agilent 2100 Bioanalyzer, followed by hybridization to Affymetrix U133 Plus 2.0 Gene-Chips, which contains a total of 54,613 probe sets, in two batches. mRNA expression array data were obtained for all of the cell lines with no missing data and normalized on a log2 scale using GCRMA [43]. The data had been used in previous reported studies (NCBI Gene Expression Omnibus, http://www.ncbi.nlm.nih.gov/geo, SuperSeries accession number GSE24277) [39]–[41]. The mean and standard deviations (SD) were calculated for each mRNA expression probe set with the GCRMA normalized values. Outliers with mRNA expression values more than 4 SD from the mean expression value were replaced with the maximum outlier value (mean expression value +/−4 SD). Similar to the genotype data, prior to GSA the expression values were adjusted using the same model including the race effect, population stratification eigenvectors, gender, and batch effect.

### Gene set eQTL association analysis (GS-eQTL)

SNPs within 20 kb of the flanking sequence of a gene were mapped to the gene, with multiple SNP-to-gene mappings allowed. mRNA expression probe sets were also mapped to their respective genes. All PharmGKB [32] and KEGG [31] GSs were downloaded and genes mapped to GSs with multiple gene-to-GS mappings allowed (http://www.pharmgkb.org/, http://www.genome.jp/kegg/). For GSs containing SNP genotypes (GS_SNP_) or mRNA expression values (GS_expression_), we define a “*cis*-GS” association to reference an association between SNPs and expression probe sets that mapped to the same GS (GS_SNP_  =  GS_expression_). A “*trans*-GS” association is defined to represent the association between SNPs and expression probe sets that mapped to different GSs (GS_SNP_ ≠ GS_expression_). Hierarchical clustering using *hclust*, an R function [44], was used to visualize the overlap existing between the PharmGKB and KEGG GSs. We defined a distance measure between GSs to be 1–τ, where τ represents the average proportion of genes shared between the GSs.

With SNPs and expression probes mapped to GSs, we sought to model the association between all GS_SNP_ and GS_expression_ within PharmGKB and KEGG GSs using a multivariate linear model. Let GS_SNP_ and GS_expression_ represent all the adjusted SNP genotypes and expression probe set values, respectively, mapped to genes contained in a GS. For each set of SNPs within the given GS, GS_SNP,_ we performed a principle component analysis (PCA) to reduce the dimensionality of GS_SNP_ [45]. This approach has been applied with success in other GSA methods to produce a lower-dimensional GS [46], [47]. In addition, PCA is a commonly used approach for modeling the association of multiple SNPs within a gene, as opposed to GS [38], [48]. The design matrix was then constructed using the components that explain 80% of the variance of the adjusted SNP genotypes within the GS of interest (i.e., design matrix of predictors variables is defined as **X**  =  PCA_80%_(GS_SN*P*_)). Similarly, PCA is also applied to GS_expression_, where we also keep the components that explain 80% of the variance of the adjusted mRNA expression values (i.e., response variable is defined as **Y**  =  PCA_80%_(GS_expression_)).

Next, we define the GS-eQTL model as **Y**  =  **B**_0_ + **B**_1_*X + **ε**, where **B**_1_ represents the vector of SNP effects (represented by the principal components needed to explain 80% of the variation), **B**_0_ represents the intercept and **ε** is the error assumed to follow a normal distribution with mean zero and common variance, N(0,σ^2^). The test of association between the expression and SNP GSs is then completed by assessing **B**_1_ using a multivariate analysis of variance with a Wilk's lambda test statistic where under the null hypothesis this vector of effects equals zero (H_0_: **B**_1_ = **0**). To account for the multiple testing and correlation between GS tests we computed false discovery rates (FDR) using 10,000 permutations [49]. Permutations were completed by shuffling the samples' expression values while holding the SNP data fixed and re-performing all GS-eQTL analyses for each permutation.

### Replication of top two GS-eQTL results

Replication of two KEGG GS-eQTL (one *cis* and one *trans*) associations detected in the analysis of SNP and mRNA expression data measured on the Coriell cell lines was completed using publically available data on the HapMap cell lines. SNP data was downloaded for the Phase 2 HapMap CEU (unrelated) cell lines, while gene expression data was downloaded from the Gene Expression Omnibus (GEO) for GEO7792 (http://www.ncbi.nlm.nih.gov/geo/query/acc.cgi?acc  =  GSE7792). GS-eQTL analyses were completed in a similar fashion as outlined for the GS-eQTL analysis of the Coriell cell line model system mRNA expression and SNP data.

## Results

### Gene set mapping

A total of 60 and 201 GSs were downloaded from PharmGKB and KEGG databases, respectively, and were used to map SNPs and expression probe sets measured on the LCLs. **Table** **1** summarizes the total number of genes, SNPs and expression probe sets mapped to PharmGKB and KEGG GSs, as well as, GS sizes and amount of gene overlap between different GSs for both resources. For PharmGKB the GS sizes ranged from 2–64 genes with an average size of 14 genes compared to a range of 1–1100 genes with an average of 70 genes for KEGG GSs. The number of genes overlapping between different GSs was also larger for KEGG GSs as compared to PharmGKB GSs. When only considering GSs with overlapping genes, KEGG averaged a 10.5 gene overlap while PharmGKB averaged a 2.7 gene overlap.

**Table 1:** Summary of gene expression and SNP GS mappings for PharmGKB and KEGG.

Source	Total genes mapped	Genes per GS			Gene overlap			SNPs mapped		Expression probe sets mapped	
		Avg.	Max	Min	Avg.	Max	Min	Max	Min	Max	Min
PharmGKB	511	13.93	64	2	0.76	18	0	4384	172	192	2
KEGG	5333	70.02	1100	1	1.96	126	0	50871	35	2149	1

**Figure 1:**
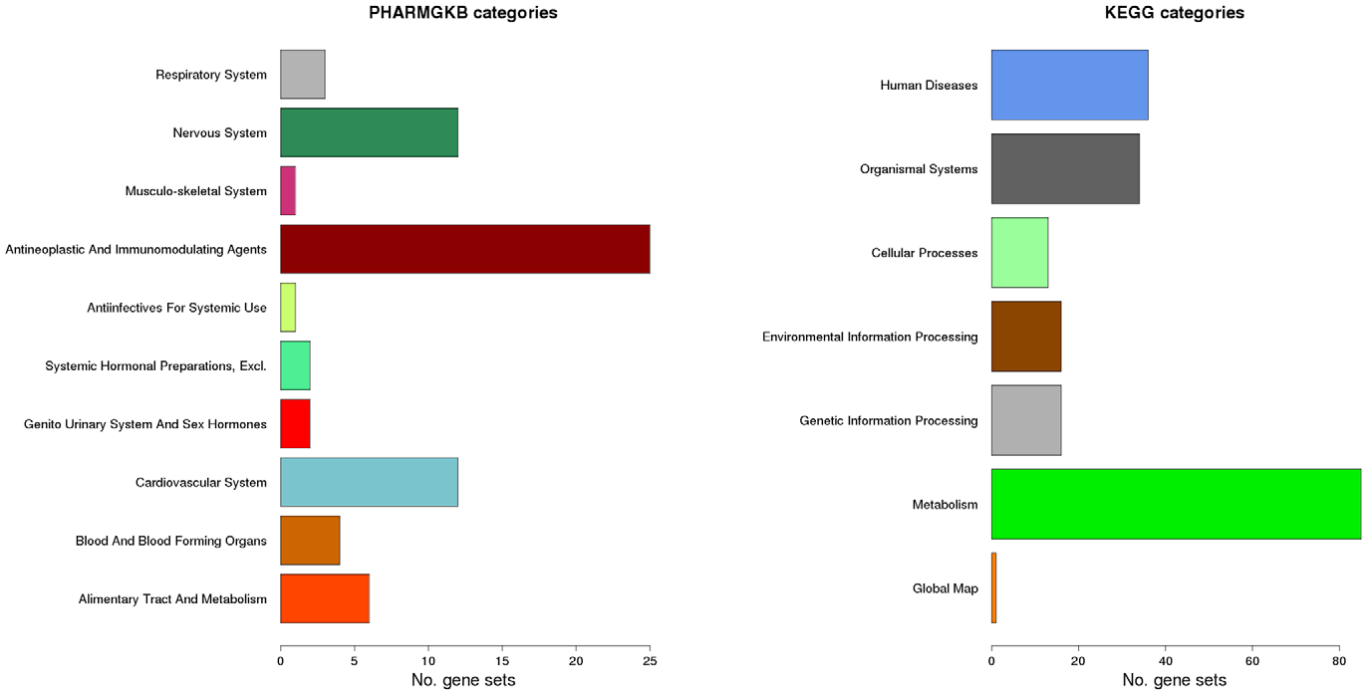
Barplots for number of GSs in PharmGKB and KEGG categories.

General GS categories and sub-categories (KEGG only) were also identified for PharmGKB and KEGG GSs (**Table** **S1**). The PharmGKB categories designate different therapeutic groups, while the KEGG categories delineate biological functions or areas to classify the GSs. For the PharmGKB and KEGG GSs used in our analysis, there were 10 and 7 GS categories ranging between 1 and 25 and 1 and 85 GSs, respectively (**Figure** **1**).

### Top GS-eQTL associations

There were 436 PharmGKB GS associations between overall genetic and gene expression variation with FDR values <0.05. The top 20 PharmGKB GS-eQTL associations are presented in **Table** **2** **.** For KEGG GSs, there was a large number of highly significant GS associations (minimum nominal p = 3.84x10^−129^); however, the majority of the top results were driven by the 13 GSs which include the human leukocyte antigen (HLA) genes. Among the top 100 results (nominal p<10^−40^), only 7 involved GSs without HLA genes. After removing these HLA genes from the GS-eQTL analysis for the KEGG GSs, there were 28161 GS associations with FDR values <0.05. The top 20 KEGG GS-eQTL associations are presented in **Table** **3** **.**

**Table 2:** Top 20 PharmGKB GS-eQTL associations.

Type	GS_expression_				GS_SNP_				GS-eQTL p-value	FDR
	Gene Set	No. Genes	No. Probe sets	No. PCs	Gene Set	No. Genes	No. SNPs	No. PCs		
cis	VEGF Pathway	15	41	9	VEGF Pathway	15	700	57	1.4×10^−23^	0
trans	EGFR Inhibitors Pathway PD	64	192	26	VEGF Pathway	15	700	57	1.4×10^−12^	0
trans	Thiopurine Pathway	32	82	14	Antiarrhythmic Drug Pathways	55	3667	143	5.1×10^−11^	0
cis	Glucocorticoid and Inflammatory genes Pathway PD	9	31	6	Glucocorticoid and Inflammatory genes Pathway PD	9	571	31	1.5×10^−10^	0
cis	Methotrexate Pathway	29	74	16	Methotrexate Pathway	29	2415	87	3.9×10^−10^	0
cis	EGFR Inhibitors Pathway PD	64	192	26	EGFR Inhibitors Pathway PD	64	4384	130	3.9×10^−10^	0
trans	Fluoropyrimidine PK	24	62	12	Antiarrhythmic Drug Pathways	55	3667	143	7.7×10^−10^	0
trans	EGFR Inhibitors Pathway PD	64	192	26	Antiarrhythmic Drug Pathways	55	3667	143	7.9×10^−9^	1.1×10^−5^
cis	Etoposide Pathway	12	33	4	Etoposide Pathway	12	1104	47	4.3×10^−8^	3.8×10^−5^
cis	Bisphosphonate Pathway	19	55	13	Bisphosphonate Pathway	19	775	51	4.9×10^−8^	4.0×10^−5^
trans	Bisphosphonate Pathway	19	55	13	Antiarrhythmic Drug Pathways	55	3667	143	5.1×10^−8^	4.0×10^−5^
trans	Fluoropyrimidine PK	24	62	12	Methotrexate Pathway	29	2415	87	7.7×10^−8^	4.9×10^−5^
cis	Statin Pathway Cholesterol and Lipoprotein Transport PD	26	55	11	Statin Pathway Cholesterol and Lipoprotein Transport PD	26	1286	83	8.0×10^−8^	4.9×10^−5^
trans	Selective Serotonin Reuptake Inhibitors SSRI Pathway	28	72	12	Antiarrhythmic Drug Pathways	55	3667	143	9.9×10^−8^	5.2×10^−5^
cis	Fluoropyrimidine PK	24	62	12	Fluoropyrimidine PK	24	2483	83	1.1×10^−7^	5.2×10^−5^
trans	EGFR Inhibitors Pathway PD	64	192	26	Selective Serotonin Reuptake Inhibitors SSRI Pathway	28	1975	98	1.2×10^−7^	5.2×10^−5^
cis	Nicotine PD Pathway Dopaminergic Neuron	20	42	9	Nicotine PD Pathway Dopaminergic Neuron	20	1049	74	1.9×10^−7^	6.3×10^−5^
trans	Methotrexate Pathway	29	74	16	Antiarrhythmic Drug Pathways	55	3667	143	2.0×10^−7^	6.3×10^−5^
cis	Antiarrhythmic Drug Pathways	55	127	14	Antiarrhythmic Drug Pathways	55	3667	143	2.9×10^−7^	9.3×10^−5^
trans	VEGF Pathway	15	41	9	EGFR Inhibitors Pathway PD	64	4384	130	4.1×10^−7^	0.0001

**Table 3:** Top 20 KEGG GS-eQTL associations after removing the genes within the HLA region.

Type	GS_expression_				GS_SNP_				GS-eQTL p-value	FDR
	Gene Set	No. Genes	No. Probe sets	No. PCs	Gene Set	No. Genes	No. SNPs	No. PCs		
cis	Metabolic pathways	1100	2149	67	Metabolic pathways	1100	50871	189	7.9×10^−85^	<5×10^−8^
trans	Metabolic pathways	1100	2149	67	Neuroactive ligand receptor interaction	302	14725	173	2.6×10^−58^	<5×10^−8^
trans	Metabolic pathways	1100	2149	67	Calcium signaling pathway	178	12520	172	5.3×10^−50^	<5×10^−8^
trans	Metabolic pathways	1100	2149	67	Pathways in cancer	330	18667	175	1.1×10^−45^	<5x10−^8^
trans	Metabolic pathways	1100	2149	67	Vascular smooth muscle contraction	125	8588	162	5.9×10^−45^	<5x10^−8^
trans	MAPK signaling pathway	273	683	48	Metabolic pathways	1100	50871	189	7.5×10^−43^	<5×10^−8^
trans	Metabolic pathways	1100	2149	67	Cytokine cytokine receptor interaction	278	9377	162	1.1×10^−42^	<5×10^−8^
trans	Metabolic pathways	1100	2149	67	Focal adhesion	201	12846	169	7.3×10^−42^	<5×10^−8^
trans	Pathways in cancer	330	893	50	Metabolic pathways	1100	50871	189	1.3×10^−39^	<5×10^−8^
trans	Metabolic pathways	1100	2149	67	Axon guidance	129	9757	163	2.8×10^−39^	<5×10^−8^
trans	Metabolic pathways	1100	2149	67	Dilated cardiomyopathy	92	7964	155	4.5×10^−39^	<5×10^−8^
trans	Metabolic pathways	1100	2149	67	Chemokine signaling pathway	190	9422	157	7.5×10^−39^	<5×10^−8^
cis*	Pathways in cancer	330	893	50	Pathways in cancer	330	18667	175	4.9×10^−38^	<5×10^−8^
Trans	Metabolic pathways	1100	2149	67	Neurotrophin signaling pathway	126	6436	146	2.1×10^−37^	<5×10^−8^
Trans	Metabolic pathways	1100	2149	67	Arrhythmogenic right ventricular cardiomyopathy ARVC	76	8274	158	2.7×10^−37^	<5×10^−8^
Trans	Metabolic pathways	1100	2149	67	Hypertrophic cardiomyopathy HCM	89	7492	154	3.2×10^−37^	<5×10^−8^
Trans	Metabolic pathways	1100	2149	67	Purine metabolism	159	9165	159	7.2×10^−37^	<5×10^−8^
trans*	Pathways in cancer	330	893	50	Neuroactive ligand receptor interaction	302	14725	173	1.9×10^−36^	<5×10^−8^
trans	MAPK signaling pathway	273	683	48	Pathways in cancer	330	18667	175	2.1×10^−36^	<5×10^−8^
trans	Metabolic pathways	1100	2149	67	GnRH signaling pathway	101	6256	152	4.1×10^−36^	<5×10^−8^

* GS-eQTL associations assessed in HapMap for replication.

**Figure 2:**
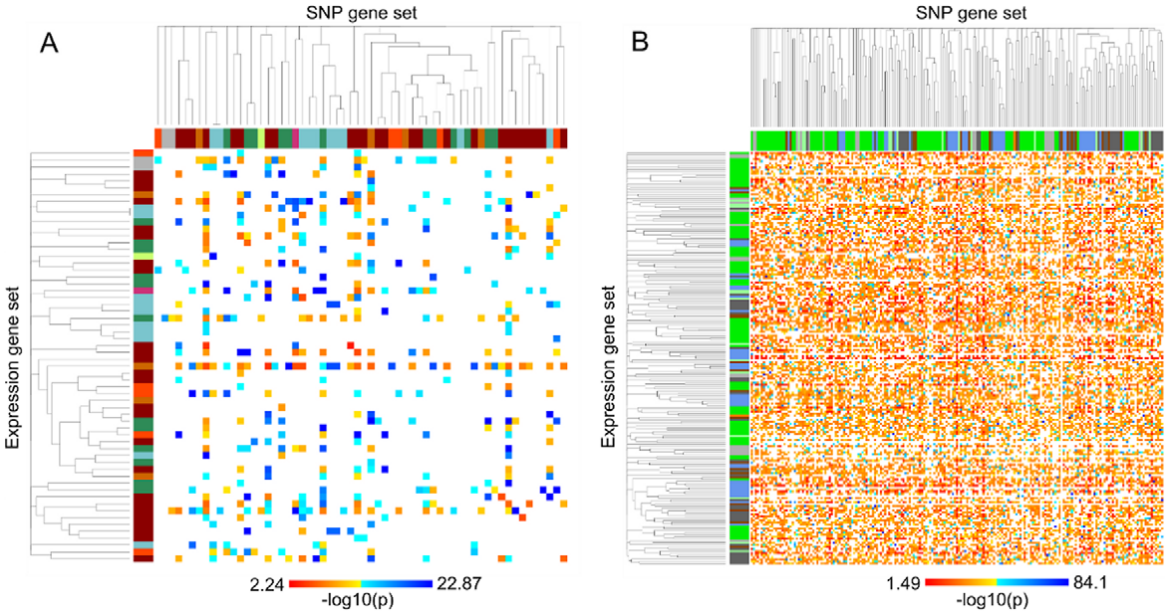
Heatmaps with points indicating associations (FDR <5%) between SNP (x-axis) and expression (y-axis) GSs. SNP and expression GSs are indexed based on hierarchical clustering using distances between GSs (distance determined by average proportion of genes shared between GSs). The color of the points indicate the level of association significance (blue  =  less significant, red  =  more significant)

For both the PharmGKB and KEGG GSs, *cis*-GS associations were the most significant: PharmGKB “VEGF pathway” (p = 7.46×10^−18^) and KEGG “Metabolic pathways” (nominal p = 7.86×0^−85^). All PharmGKB and KEGG GS associations with FDR<0.05 are displayed in the heatmaps (**Figure 2**), with SNP and expression GSs indexed on the x- and y-axis, respectively. The SNP and expression GSs are ordered on the axes by the order established using hierarchical clustering with distances between GSs, where distance is 1 – τ (τ  =  average proportion of genes shared between GSs). The clusters are shown on the left and upper axes in **Figure 2**, with the colors indicating the GS categories. While the average “distance” between different GSs for PharmGKB and KEGG are 0.97 and 0.91, respectively, there are clusters of GSs due to overlaps of genes. However, **Figure 2** does not indicate a strong clustering among GSs within the same category, with a lower average distance between GSs within the same category as compared to the average distance between GSs in different categories (PharmGKB  = 0.47 verses 0.97; KEGG  = 0.49 verses 0.98).

**Figure 3:**
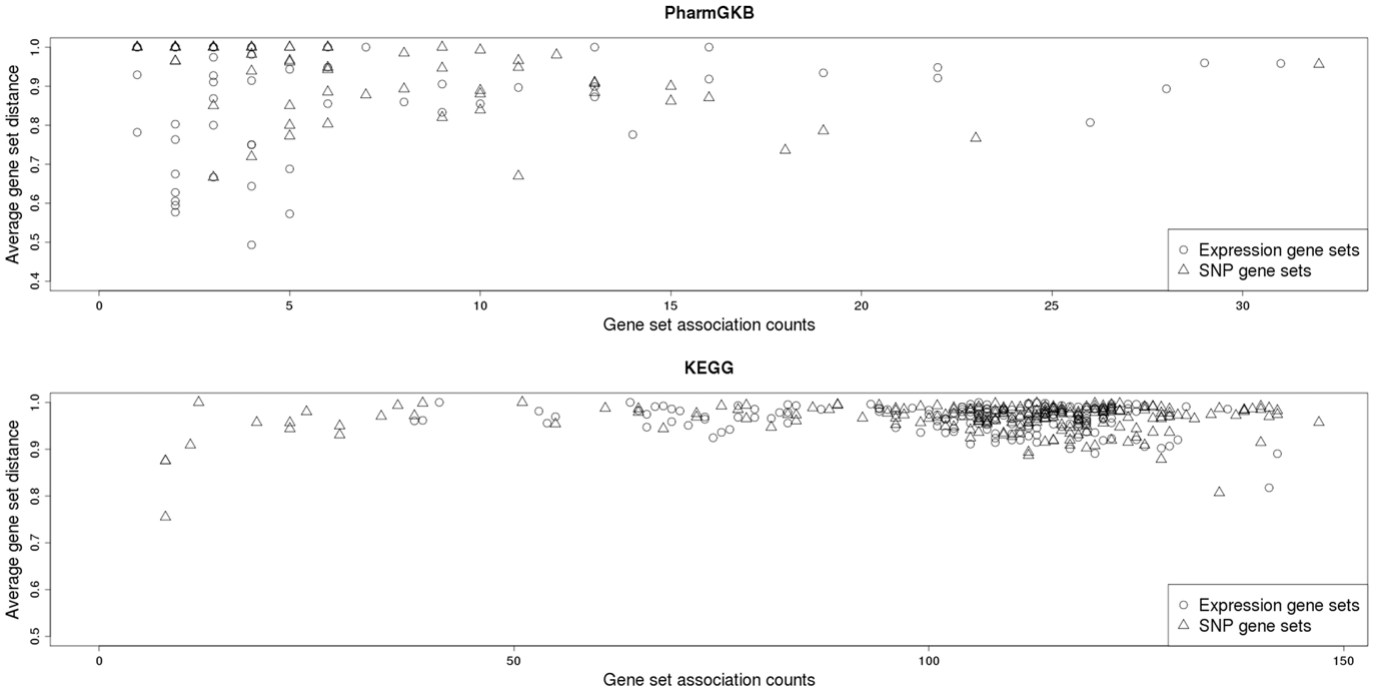
Scatter plot of the number of associations (FDR <5%) each GS was involved in against the average distance between each GS and GSs associated with it.

**Figure 2** also provides a visual for the GSs which are involved in a large number of significant associations, either as a SNP or expression GS. The highly associated SNP GSs appear as vertical lines in the heatmaps, indicating their association with a large number of expression GSs, while the highly associated expression GSs similarly appear as horizontal lines. **Table 4** lists the five SNP and expression GSs involved in the most GS associations for PharmGKB and KEGG. For PharmGKB, “EGFR Inhibitors Pathway PD” had the most associations (31 associations) as an expression GS, while “Antiarrhythmic Drug Pathways” had the most associations (32 associations) as a SNP GS. For the analysis of the KEGG GSs, the expression GS involved in the most associations was “Pathways in cancer” (142 associations), while the “Calcium signaling pathway” was involved in the most associations (142 associations) as a SNP GS. **Figure 3** is a plot of the number of associations for each GS against each GS's average distance (based on the proportion of overlap of genes) to the GSs associated with it. The five highly associated GSs for PharmGKB and KEGG all have average distances >0.87, indicating that the large number of associations is not simply due to an overlap between GSs.

**Table 4:** Top five SNP and expression GSs involved in the most associations (FDR <5%).

Source	Data type	Gene Set	No. significant associations
		EGFR Inhibitors Pathway PD	31
		Selective Serotonin Reuptake Inhibitors SSRI Pathway	29
	Expression	Methotrexate Pathway	28
		Doxorubicin Pathway	26
PharmGKB		Antiarrhythmic Drug Pathways	22
		Antiarrhythmic Drug Pathways	32
		Taxane Pathway	23
	SNP	Imatinib	19
		Doxorubicin Pathway	18
		Fluoropyrimidine PK	16
		Pathways in cancer	142
		Metabolic pathways	141
	Expression	Cysteine and methionine metabolism	136
		ABC transporters	131
KEGG		Insulin signaling pathway	130
		Calcium signaling pathway	147
		Tyrosine metabolism	142
	SNP	Ether lipid metabolism	142
		Nucleotide excision repair	141
		Antigen processing and presentation	141

**Figure 4:**
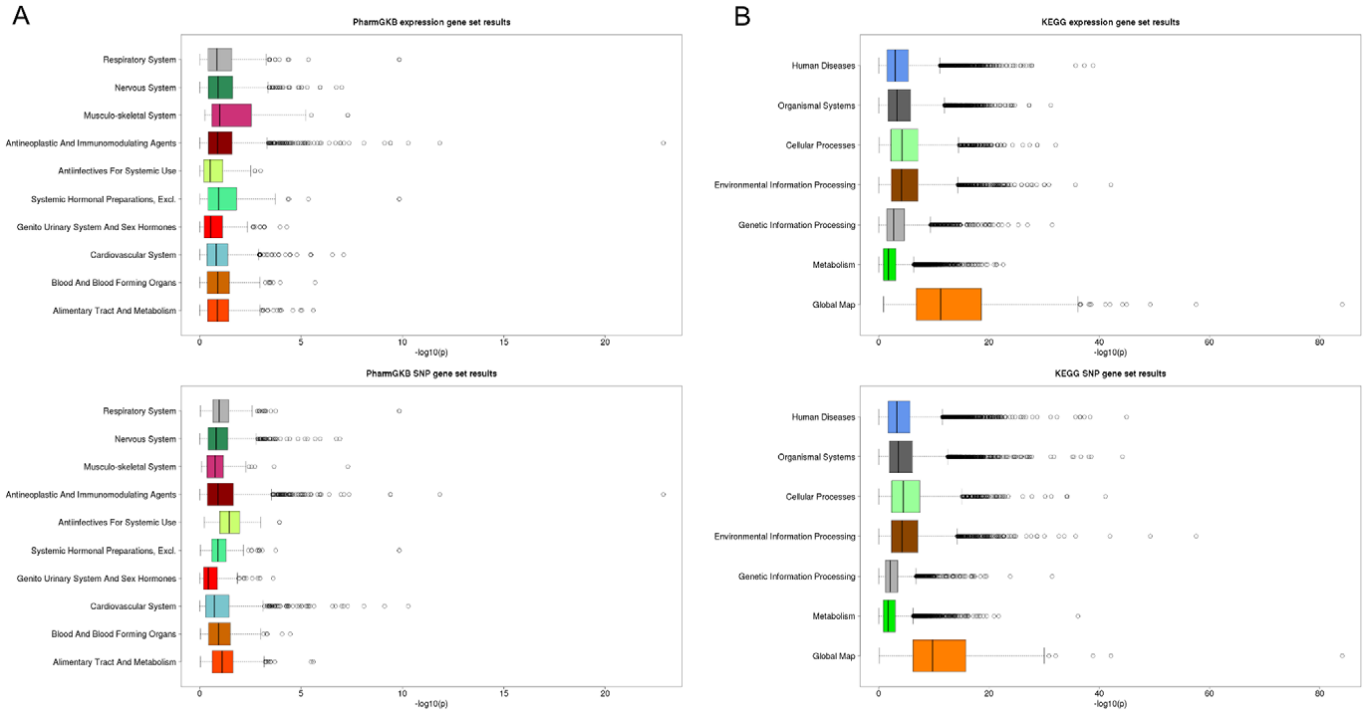
Boxplots of log transformed p-values for GS-eQTL association results by category. (A) PharmGKB and (B) KEGG.

Next, **Figure 4** shows boxplots of the log transformed p-values for all SNP and expression GS associations by GS category. The largest PharmGKB GS category, Antineoplastic and Immunomodulating Agents, also contained the most significant association, a *cis*-GS association for the “VEGF pathway” (nominal p = 7.46×10^−18^). The KEGG category, Global Map, contained the most significant *cis*-GS association for the GS “Metabolic pathways” (nominal p = 7.86×10^−85^). Comparing the SNP and expression GS associations by PharmGKB and KEGG categories, little differences were observed. The level of association results also appeared to be evenly distributed amongst the categories, other than the KEGG Global Map category having many more highly significant associations than the other categories for KEGG.

### Cis- verses trans- gene set associations

**Figure 5:**
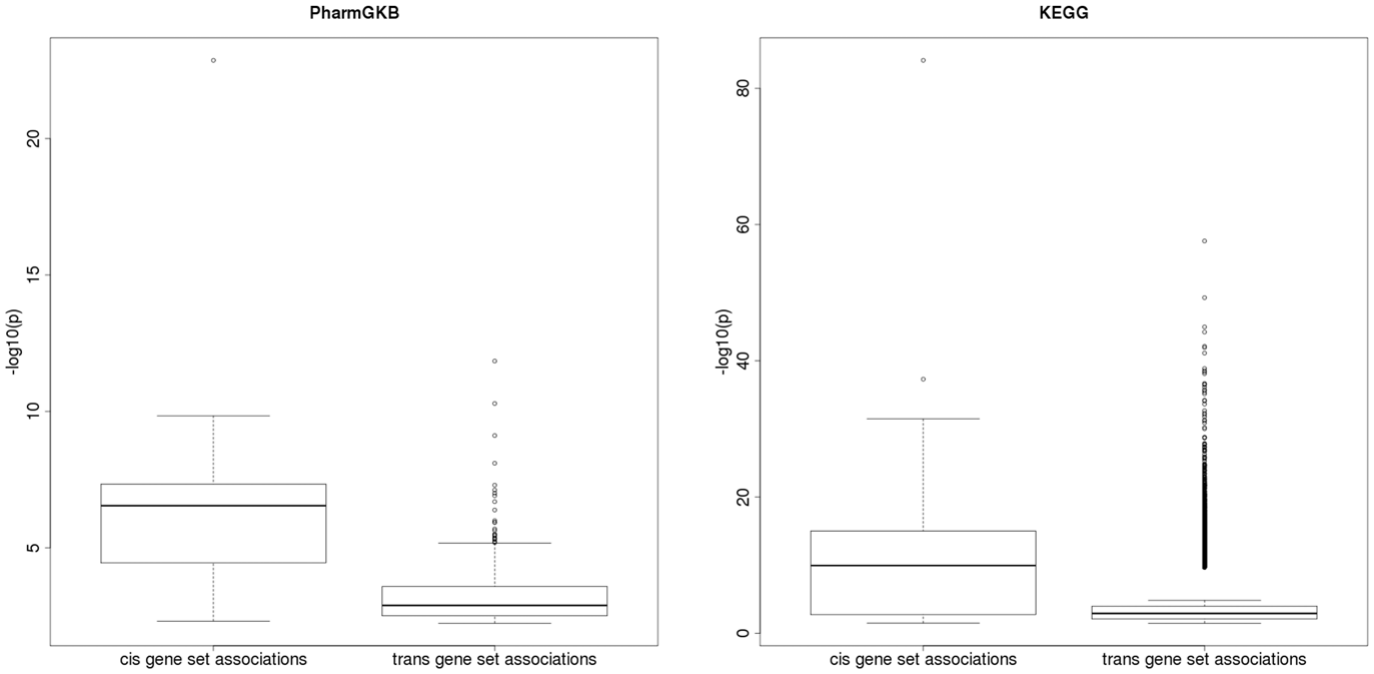
Boxplots of log transformed p-values for *cis*- and *trans*-GS association results. (A) PharmGKB and (B) KEGG.

To assess if we observed more *cis*- or *trans*- GS associations for both the PharmGKB and KEGG GSs, we tested whether a disproportionate amount of *cis*- or *trans*-GS associations among the findings with FDR <5% were observed. For the PharmGKB associations with FDR <5%, there were 19 out of 60 (32%) *cis*- and 417 out of 3,540 (11.8%) *trans*-GS associations (empirical p<4.0×10^−4^). In contrast, for the KEGG GS-eQTL analysis, 188 out of 201 (94%) *cis*-GS associations had FDR <5% while and 27,973 out of 40,200 (70%) *trans*-GS associations had FDR <5% (empirical p<1.0×10^−4^). **Figure 5** illustrates this larger number of significant GS associations for *cis*-relationships as compared to *trans*-relationships for both PharmGKB and KEGG GS-eQTL analyses.

### Replication of top two GS-eQTLs

Replication of the top KEGG GS-eQTL associations involving the Pathways in Cancer and Neuroactive ligand-receptor interaction was completed using publically available SNP and mRNA expression data measured on the CEU HapMap LCLs. The analysis *cis* GS-eQTL association between the Pathways in Cancer GS in the HapMap data resulted in a p-value of 0.00076. Similarly, the *trans*-association between the variation in mRNA expression levels for genes within the Pathways in Cancer GS and the genetic variation for genes within the Neuroactive ligand-receptor interaction GS was replicated with a p-value of 0.036.

## Discussion

Using SNP and expression genome-wide data collected on a cell based model system we applied a new approach, GS-eQTL analysis, to identify genetic variation associated with mRNA gene expression in the context of GSs or pathways. By modeling the genetic variation and expression using GSs we were able to increase statistical power by reducing the multiple testing inherit with high dimensional genomic data and combining the genetic variation and mRNA expression of functionally related genes, as defined by PharmGKB and KEGG. GSs have recently been used in a variety of settings for increased power [50]–[53]; however, limited research has been completed to apply the ideas of GSA to the study the genetics of gene expression and eQTL analysis.

After adjusting for multiple testing, we determined a large number of significant GS-eQTL associations (FDR <5%) for both GSs in PharmGKB and KEGG. Replication was attempted for two of the top association for KEGG GSs using the publically available data on the HapMap samples. The “Pathways in Cancer” *cis* GS-eQTL and the *trans* GS-eQTL association between the variation in mRNA expression levels for genes within the “Pathways in Cancer” GS and the genetic variation for genes within the “Neuroactive ligand-receptor interaction” GS were both replicated using the publically available data from HapMap with p-values of 0.00076 and 0.036, respectively. The first canonical correlation between the mRNA gene expression and SNP genotypes for “Pathways in Cancer” *cis* GS-eQTL and the *trans* GS-eQTL association between the “Pathways in Cancer” and the “Neuroactive ligand-receptor interaction” GSs was 0.98 for both GS-eQTLs.

Examining all pairwise SNP-expression associations within the “Pathways in Cancer” found an association between rs2235529 within *WNT4* and the mRNA expression level of *CDC42* (208727_s) with a p-value of 1.61×10−42 (Bonferroni correction for testing all pairwise associations within this GS results in a p-value  = 3.9×10^−35^). These two genes are 25 kb apart, suggesting a typical *cis* regulatory relationship. There were an additional 31 eQTL associations within “Pathways in Cancer” GS with Bonferroni adjusted p-values <1×10^−9^, with *cis* associations observed for 5 genes within the GS. For the second replicated GS-eQTL between the “Pathways in Cancer” GS and the “Neuroactive ligand-receptor interaction” GS, the most significant eQTL association involved SNP rs1160198 from gene *GLRA2* and expression of *IGF1R* (Bonferroni adjusted p-value of 7.95×10^−8^).

The large number of associations identified for KEGG may be due to the correlation structure that exists among the KEGG GSs or “master” regulating genes or GSs. Grouping the significant GS associations by category did not show a large difference between categories in terms of strength of association. There were also GSs involved in many GS associations either as a SNP or expression GS, which are analogous to eQTL “hotspots” in previous literature [4], [54], [55]. The SNP GSs with many associations can be considered “master regulator” GSs in terms of regulating the expression of other GSs, while expression GSs with many associations appear to be regulated by many different GSs. The concept of “master regulator” GSs may not be as straightforward as a single gene “master regulator” in a biological sense, but the GS associations may be indicating the interaction or regulation between many components involved in a complex system of biological processes or functions. Among the top findings for both GS resources, we also observed a greater proportion of *cis* GS-eQTL associations as compared to *trans* GS-eQTL associations, as one would expect from previous eQTL research [56].

Given the use of functionally defined GSs to perform GS-eQTL analysis, there are broader implications from these findings to consider beyond the standard SNP verses expression analyses, particularly for the regulatory function and/or regulation of drug pharmacokinetic (PK) and pharmacodynamic (PD) pathways. The PK and PD pathways are well characterized and studied pathways, and are composed of the elements involved in either the metabolism (PK) or targeted action (PD) of drugs. Thus, further understanding of these pathways has significant clinical impact. The *trans* GS-eQTL associations provide hypotheses to further pursue regarding the genetics of gene expression. For example, one of the top *trans*-GS associations for PharmGKB involved the expression of the “Thiopurine” pathway and the genetic variation of the “Anti-arrhythmic Drug” pathway. While these two pathways are curated for two completely different drugs, their genetic components appear to be associated.

Similarly with the KEGG results, there were many *trans*-GS associations which suggest novel hypotheses to be further explored. From the top 30 KEGG results, 25 involved the expression or genetic variation of the KEGG GS “Metabolic pathways,” indicating a significant role for these genes in the genetics of human mRNA expression. Due to the non-specific nature of many GSs, other methods, such as gene level tests, will be needed to follow up on these initial findings to determine the potential “drivers” of these associations. Thus, this method is highlighted as an effective first step to help focus follow up association and/or functional studies to establish novel associations between genome-wide genetic sequence variation and mRNA gene expression.

The use of human cell lines from unrelated subjects (i.e. lymphoblastoid cell lines from HapMap samples) for eQTL studies have recently been successful in identifying many significant findings [24], [57]; however, tissue-dependent patterns of gene expression may limit the generalization of our findings. A recent study suggests little eQTL overlap between tissues [58], while other work has found a more substantial eQTL overlap exists across tissues when considering sample size differences between eQTL studies [59]. Nonetheless, tissue dependent gene expression could play a considerable role in the context of our approach, especially when examining certain PK pathways that involve many genes that encode metabolic enzymes which are highly expressed in the liver. Future work is needed to consider GS-eQTLs studies where mRNA is measured in diverse tissue types, such as liver and adipose tissues.

In this manuscript, we focused on GS-eQTL analysis between GSs and pathways contained within PharmGKB and KEGG with SNPs mapped to within 20 kb of the 3′ and 5′ ends of each gene. Considering variation beyond 20 kb may include more functional variants, but studies have shown that much of the key variation lies within 20 kb of the gene transcription start and end sites [60]. Additionally, the current definitions of PharmGKB and KEGG pathways are incomplete and have a clear bias towards studies involving certain genes and therapeutic agents, and thus limit the scope of our conclusions. However, the novel GS-eQTL analysis proposed has the ability to easily be extended to other pathway or GS sources such as Gene Ontology (GO) [30].

Application of PCA in our GS-eQTL analysis method effectively reduced the dimensionality of the genomic data. However, in applying PCA one must deal with missing data. In our analysis, we removed SNPs with a call rate <95%. Due to the small amount of missing genotypic data, we chose to impute the mean SNP genotype (in terms of the number of minor alleles) for missing genotypes. Another approach to deal with missing genotypic data would be to use one of the various genotype imputation methods [61]. A second limitation is that PCA only assesses linear relationships as a means of dimension reduction between the data which may not be optimal for all GSs. Future work is on-going to determine an approach to reduce the dimensionally of the genetic and mRNA expression data using both linear and non-linear relationship, such as kernels [62], [63], along with the application of this approach to other forms of genomic data, such as microRNA or methylation data.

In conclusion, we have demonstrated an efficient approach to analyze the high dimensional data for studying the genetics of gene expression with application of the GS-eQTL approach to determine novel relationships between GSs and pathways within PharmGKB and KEGG. A systems biology approach with GSs is a natural application towards studying the genetics of gene expression to reduce the high-dimensionality of the data and to make use of GSs grouped based on a biological process or function in which there already may be an expected relationship between the annotated GS processes or functions. Developing and applying new approaches, such as ours, to analyze the high-dimensional genomic data to identify associations is a necessary step towards establishing the regulatory relationships at the molecular level, which will help translate findings from disease risk or pharmacogenomic studies towards meaningful biology.

## Supporting Information

Table S1 **Categories of Gene Sets.**
(XLSX)
